# Persistent depressive disorder across the adult lifespan: results from clinical and population-based surveys in Germany

**DOI:** 10.1186/s12888-020-2460-5

**Published:** 2020-02-10

**Authors:** Julia Nübel, Anne Guhn, Susanne Müllender, Hong Duyen Le, Caroline Cohrdes, Stephan Köhler

**Affiliations:** 1 Department of Epidemiology and Health Monitoring, Unit 26 Mental Health, Robert Koch Institute, PO Box 650261, 13302 Berlin, Germany; 2 Department of Psychiatry and Psychotherapy, Charité – Universitätsmedizin Berlin, Campus Mitte, Charitéplatz 1, 10117 Berlin, Germany

**Keywords:** Chronic depression course, Persistent depressive disorder, Prevalence, Disease burden, Health-related quality of life, General population, Germany, Epidemiology, Cross-sectional studies, Clinical studies

## Abstract

**Background:**

Although the individual and economic disease burden of depression is particularly high for long-term symptoms, little is known of the lifetime course of chronic depression. Most evidence derives from clinical samples, and the diagnostic distinction between persistent depressive disorder (PDD) and non-chronic major depression (NCMDD) is still debated. Thus, we examined characteristics of PDD among clinical vs. non-clinical cases, and the associated disease burden at a population level.

**Methods:**

Data were drawn from the mental health module of the German Health Interview and Examination Survey for Adults (DEGS1-MH, 2009–2012, *n* = 4483) and a clinical sample of PDD inpatients at Charité – Universitätsmedizin Berlin (2018–2019, *n* = 45). The DSM-5 definition of PDD was operationalized a priori to the study using interview-based DSM-IV diagnoses of dysthymia and major depression lasting at least 2 years in both surveys. Additional depression characteristics (depression onset, self-classified course, suicidality, comorbid mental disorders, treatment history and current depressive symptoms [Patient Health Questionnaire-9]) were assessed. In the DEGS1-MH, health-related quality of life (Short Form Health Survey-36, SF-36), chronic somatic conditions, number of sick days (past 12 months) or days with limitations in normal daily life activities (past 4 weeks), and health service utilization (past 12 months) were compared for PDD vs. NCMDD.

**Results:**

PDD cases from the clinical sample had a significantly earlier depression onset, a higher proportion of self-classification as persistent course, and treatment resistance than PDD and NCMDD cases in DEGS1-MH. At a population level, PDD cases showed worse outcomes compared with NCMDD cases in terms of somatic comorbidity, SF-36 mental component score, and activity limitations owing to mental health problems, as well as a higher risk for outpatient mental health care contact.

**Conclusions:**

The distinction between PDD and NCMDD proposed for DSM-5 seems warranted. Early onset depression, self-classification as persistent depressive course, and treatment resistance are suggested as markers of more severe and chronic depression courses. At a population level, PDD is associated with remarkably higher individual and economic disease burden than NCMDD, highlighting the need to improve medical recognition of chronic courses and establish specific treatment concepts for chronic depression.

## Background

More than 300 million people globally were affected by depression in 2015, reflecting an increase of about 18% since 2005 in clinical settings [1]. In terms of years lived with disability, depressive disorder is now a leading contributor to non-fatal health loss [2]. Owing to its individual and economic disease burden, depression has become a global core health challenge of the twenty-first century [3–7]. Social insurance agencies in Germany have reported an increase in the frequency of depression and growing health care costs owing to working days lost, early retirement, and increased health service provision [8, 9].

However, there are individual differences in depression course (i.e., single episodes vs. recurrent episodes), type, and severity. The enormous economic impact of depression on the general population seems particularly related to its duration (i.e., long-term), rather than to its severity [10–14]. Primary data indicate that up to 30% of depression cases have a chronic course with symptoms that last for at least 2 years [12, 15–17]. The 12-month prevalence of chronic depression is 1.5% [18] and its lifetime prevalence is 3 to 6% [16–18]. In Germany, there is a lack of population-based information on chronic vs. non-chronic depression courses. However, secondary data from national health insurance companies indicate that up to two-thirds of medical depression diagnoses take a chronic course over at least 2 years (repeated registration irrespective of type or severity) [19].

Furthermore, chronic depression may have an earlier onset (before 21 years of age) [14, 20–22] and worse outcomes than non-chronic depression, such as single or recurrent depressive episodes with full inter-episode recovery. Chronic depression is characterized by higher comorbidity rates [12–15, 18, 20, 22], somatic morbidity [14, 15], suicidality [14, 20, 22], reduced somatic and psychological well-being and health-related quality of life [12–14, 23], lower employment rates [24], longer delays for treatment [15], and limited effects of psychotherapeutic or psychopharmacological treatment [10, 11, 13, 25–27], all of which indicate its enormous direct and indirect costs.

However, comparisons of the characteristics, prevalence, and disease burden of chronic vs. non-chronic depression is hampered by two facts: most knowledge derives from clinical samples [15] and prevalence estimates differ, because a generally accepted definition of chronic depression was lacking until the American Psychiatric Association in 2013 decided to include a new depressive subtype, persistent depressive disorder (PDD), in the latest version of the Diagnostic and Statistical Manual of Mental Disorders, Fifth Edition (DSM-5) [28, 29]. PDD is defined as depression that persists for at least 2 years. The PDD subtype is thus a combination of the DSM-IV diagnoses of (lasting) major depressive disorder (MDD) and dysthymic disorder (DD). However, even the new PDD diagnostic category does not consider additional lifetime information [25]. Thus, little is known about chronic depression during the lifespan (e.g., regarding early vs. late onset depression). Furthermore, the DSM-5 PDD diagnosis relies predominantly on clinical data and the concept of PDD has been criticized [30], as its reliability has not been formally examined [31]. However, some researchers still argue for a diagnostic distinction between chronic and non-chronic forms of MDD [32].

In this study, we aimed to comparatively analyze and differentiate characteristics of PDD vs. non-chronic depression courses during the lifetime using population-based data from the German health monitoring program at the Robert Koch Institute and a clinical sample from Charité – Universitätsmedizin Berlin. We hoped to extend the knowledge of chronic depression beyond the data from clinical samples, provide frequency information at a population level, and quantify the individual and economic disease burden of chronic depression for the general population in Germany. The findings from clinical studies suggest that both the indirect costs (e.g., to health-related quality of life or sick (leave) days) and the direct costs of health service utilization and treatment resistance are much higher for PDD cases than for non-chronic cases.

The study objectives were 1) the classification of chronic vs. non-chronic depression courses at a population level, 2) the identification of PDD characteristics in a clinical vs. population-based sample, and 3) the comparison of PDD vs. non-chronic MDD (NCMDD) in terms of associations with health-related correlates at a population level.

## Methods

### Data basis and depression assessment

Data for the nationwide representative analyses were drawn from the first wave of the German Health Interview and Examination Survey for Adults (DEGS1, field work 2008–2011, *n* = 7115) and its mental health module (DEGS1-MH, field work 2009–2012, *n* = 4483), which included 18- to 79-year-old participants from statutory as well as private health insurances based on a two-stage clustered random sampling procedure (step 1: random sampling of study locations from all municipal communities; step 2: random sampling of participants from the population-registries in each sampled study location). The design and methods are described in detail elsewhere [33–35]. DEGS1 and DEGS1-MH were part of the German health monitoring program and provided data about the health of the non-institutionalized population in Germany based on self-rated questionnaires and a standardized computer-assisted Interview conducted by study physicians (CAPI). Mental disorders, including MDD and DD, were assessed by trained interviewers based on the World Health Organization Composite International Diagnostic Interview (CIDI). The CIDI is a standardized fully structured computer-assisted clinical face-to-face interview and is an internationally established measure of mental disorders [36–38]. A modified German version of the CIDI was used in DEGS1-MH [33] to assess mental disorders according to the DSM-IV-TR diagnostic criteria [39]. The CIDI provides lifetime information about symptoms (e.g., age of onset, recurrence and duration of episodes) that permits analysis of the course of depression over the lifespan. After participants with missing information on affective disorders were excluded (*n* = 75), the final study sample was *n* = 4408.

Data were also obtained from a clinical sample recruited at the Charité – Universitätsmedizin Berlin (*n* = 60). Patients with a professional diagnosis of PDD according to DSM-5 [28] were treated for 12 weeks with a specialized chronic depression intervention: the Cognitive Behavioral Analysis System of Psychotherapy (CBASP; [27, 40]). Patients were either directly referred from the outpatient clinic of the Charité, from inpatient wards of other hospitals from all parts of Germany, or from outpatient psychiatrists. Treatment was reimbursed by statutory health insurances. Exclusion criteria for the inpatient CBASP were a history of psychotic episodes, bipolar I or II disorders, comorbid substance dependence with less than 3 months of abstinence, severe forms of autism, and organic mental disorders. All patients treated at the ward from 2013 to 2018 were invited for a subsequent follow-up interview for the purpose of the present study. These interviews were conducted from October 2018 to March 2019 to collect lifetime information on course and type of depression and comorbid mental disorders using the Structured Clinical Interview for DSM-IV (SCID I; [41]) and self-rated questionnaires. To allow comparison with the epidemiological sample, additional questions based on the CIDI depression section were included. The final clinical study sample comprised *n* = 45 patients, aged 24–66 years.

### Definition and operationalization of (non-)chronic depression

For this study, the definition of chronic depression was based on the DSM-5 PDD diagnosis and drawn from the DSM-IV-based diagnoses of MDD or DD derived from the SCID I or CIDI. According to DSM-IV, MDD diagnosis requires the persistence of at least five out of nine depressive symptoms on nearly every day for 2 weeks or longer, of which at least one is depressed mood or decreased interest/pleasure (criterion A). Furthermore, clinically significant distress and impairment associated with these symptoms are necessary (criterion C). MDD exclusion criteria include lifetime manic/hypomanic episodes (criterion B) and depressive symptoms solely attributable to the direct physiological effects of a substance or a general medical condition (criterion D) or attributable to grief (criterion E). DD diagnosis requires depressed mood for most of the day and for at least 2 years (criterion A), and at least two out of six depression symptoms (criterion B). During the 2 years, the total recovery time should not have exceeded more than 2 months (criterion C) and the symptoms should have caused clinically significant distress or impairment (criterion H). Exclusion criteria include manic/hypomanic episodes (criterion E), symptoms owing to the direct physiological effects of a substance or a general medical condition (criterion G), or symptoms occurring during the course of a psychotic disorder (criterion F). Furthermore, DSM-IV DD diagnosis requires the absence of a major depressive episode during the first 2 years of occurrence (criterion D). However, DSM-5 no longer includes this criterion for PDD diagnosis, and MDD criteria may be continuously present for 2 years.

Thus, subjects with lifetime or 12-month MDD according to CIDI or SCID I who also report a lifetime maximum episode duration of at least 104 weeks, as well as subjects (concurrently) fulfilling the DD diagnostic criteria (irrespective of DSM-IV criterion D), were classified as lifetime PDD cases. The remaining MDD cases were categorized as non-chronic cases (NCMDD). The grouping of PDD and NCMDD was carried out a priori to the study. Cases with missing responses for maximum episode duration and missing information on diagnostic criteria of DD have been omitted. In the clinical sample, health professional-diagnosed PDD was validated via SCID I for all patients.

### Depression characteristics

Age of depression onset and the number of depressive episodes were assessed in both diagnostic interviews. History of suicidality was also assessed in both surveys based on CIDI questions about thoughts of death or suicide, suicide plans, or attempted suicide.

Subjects of DEGS1-MH and patients of the clinical sample rated their course of depression based on CIDI depression section diagram on the following categories: single episode (remitted), single episode (acute), recurring episodes, single episode with chronic course, persistent depressive course, double depression, or other.

MDD symptoms according to DSM-IV were assessed using the German version of the internationally established Patient Health Questionnaire (PHQ-9). The PHQ-9 consists of nine items assessing the presence and frequency of depressive symptoms during the past 2 weeks. Summed scores ≥10 indicate current depressive symptoms [42, 43].

The number of comorbid mental disorders (lifetime) was categorized as none, one, and at least two of the CIDI- or SCID I-based diagnoses of mental disorders during the lifetime. As some mental disorders were included in the exclusion criteria for the clinical sample, the following comorbid diagnoses were assessed: panic disorder, agoraphobia, generalized anxiety disorder, social phobia, specific phobias, obsessive–compulsive disorder, posttraumatic stress disorder, pain and somatoform disorders, substance abuse and dependence (excluding nicotine), anorexia nervosa, bulimia nervosa, and binge eating disorder.

Self-reported mental health treatment during the lifetime was assessed based on the CIDI questions in both DEGS1-MH and the clinical sample. The number of antidepressant treatments and the number of psychotherapies were each categorized as none, one, and at least two treatments. Treatment resistance was defined for cases with at least two reported antidepressant treatments, approaching the definition of Thase and Rush (medication resistance to two or more adequate trials of antidepressants) [44].

### Health-related correlates

Several health-related correlates were assessed in DEGS1-MH: self-rated health (dichotomized into fair/poor vs. good/very good/excellent) and health-related quality of life (past 4 weeks) were assessed using the German version of the Short Form Health Survey-36 (SF-36) version 2 [45, 46]). The physical component score (PCS) and the mental component score (MCS) were used as total scales with a mean value of 50 and a standard deviation of 10 (higher values indicate better health-related quality of life). The number of days with limitations in normal daily life activities owing to physical vs. mental health problems (including limitations owing to substance use) during the past 4 weeks were also assessed [see 23]. The self-reported number of sick days during the past 12 months was assessed in DEGS1 (irrespective of occupational status), as well as self-reported information on health service use during the past 12 months (number of outpatient physician visits, outpatient psychiatric or psychotherapeutic contacts, and number of nights in hospital). The number of chronic somatic conditions reported in DEGS1 was classified as none, one, and at least two of the following somatic conditions [see 47]: myocardial infarction (lifetime), chronic heart failure (lifetime), stroke (lifetime), osteoarthritis (lifetime), rheumatoid arthritis (past 12 months), osteoporosis (lifetime), gout (past 12 months), bronchial asthma (past 12 months), cirrhosis of the liver (lifetime), hepatitis (past 12 months), gastric-duodenal ulcer (past 12 months), cancer (lifetime), Parkinson’s disease (lifetime), epilepsy (past 12 months), hypertension (past 12 months), dyslipidemia (past 12 months), renal failure (lifetime), and inflammatory bowel disease (past 12 months).

### Other measures

Sociodemographic variables included sex, age, marital status, and educational level. Age was assessed in years at the time of the clinical follow-up as well as of the DEGS1 mental health module assessment and categorized into age groups (18–34, 35–49, 50–64, and 65–79 years). Marital status was dichotomized into married and living with partner vs. married and not living with partner/single/never been married/divorced/widowed. The Comparative Analysis of Social Mobility in Industrial Nations (CASMIN) scale was used to classify responses on educational level into low, medium, and high. In DEGS1-MH, structural social support was assessed using the Oslo-3 Social Support Scale [48].

### Statistical analysis

Frequency and mean estimates of the sample characteristics are reported with 95% confidence intervals (95% CI).

At a population level, prevalence estimates for lifetime MDD and DD are reported. Conditional frequencies for chronic vs. non-chronic courses among lifetime MDD cases are reported. Prevalence estimates for PDD and NCMDD could not be provided owing to many missing responses for self-reported maximum episode duration, resulting in a high proportion of MDD with unknown chronicity.

Frequency and mean estimates for depression characteristics are reported with 95% CI for PDD cases in the clinical sample and for PDD and NCMDD cases in the population-based sample. The significance (*p* < .01) of differences between the clinical sample and the DEGS1-MH sample was indicated by non-overlapping 95% Cis [47] and sizes of significant effects for independent groups with different sample size are indicated by Cohen’s d (small = 0.2, medium = 0.5, large = 0.8). Statistical significance of differences between PDD and NCMDD characteristics in DEGS1-MH were evaluated using the Rao–Scott chi-square test for categorical variables, and the Wilcoxon–Mann–Whitney test for continuous variables, using a two-sided significance level of 0.05.

Health-related correlates are shown for DEGS1-MH PDD vs. NCMDD cases with 95% CI, to enable the comparison of the associated individual and economic disease burden at a population level. Effect estimates for health-related correlates in cases with PDD vs. NCMDD were based on logistic, linear, negative binomial, or zero-inflated negative binomial regression models, including health-related correlates as dependent variables and depression course (PDD vs. NCMDD) as the independent variable (reference: NCMDD). All analyses were adjusted for sex, age group, educational level, marital status, social support, chronic somatic conditions (except for analysis of the number of chronic somatic conditions as an outcome variable), and PCS (except for analysis of PCS as an outcome variable) [see 49]. The results of the unadjusted regression analyses are included as supplementary data (see Additional file 1) and only described if divergent. Statistical significance was evaluated based on a two-sided significance level of 0.05.

All statistical analyses were performed using Stata 15.1 (StataCorp, College Station, Texas, USA). For DEGS1-MH, all analyses were performed using the Stata survey design procedures to account for clustering and weighting of the study sample. Thus, survey-specific weighting factors were used to adjust the sample to the demographic distribution of the population in Germany as on 31st December, 2010, regarding sex, age, educational status, federal state, nationality, and the probability of participation in the mental health module subsequent to the core survey [33, 50].

In addition, we calculated post-hoc power analyses to test for appropriate test power based on the present sample sizes.

## Results

### Sample characteristics

Sample characteristics of the clinical sample and DEGS1-MH sample are shown in Table 1. The DEGS1-MH sample was comparable to the clinical sample on age and sex, except for the proportion of participants aged 50–64 years (higher in the clinical sample) and 65–79 years (higher in DEGS1-MH participants). Clinical sample patients more frequently lived alone (88.9% vs. 39.2%) and demonstrated a significantly higher educational level than the DEGS1-MH sample (as indicated by non-overlapping 95% Cis).

**Table 1:** Sample characteristics of the clinical and the population-based sample

	Clinical sample^1^ (*n* = 45)	DEGS1-MH^2^ (*n* = 4408)	Cohen’s *d*
Sex, % (95% CI)			
Male	53.3 (37.8–66.7)	49.1 (47.1–51.0)	0.84
Female	46.7 (33.3–62.2)	50.9 (49.0–52.9)	0.84
Age, mean (95% CI)	47.1 (43.6–50.6)	48.0 (47.4–48.6)	0.18
Age group (years), % (95% CI)			
18–34	22.2 (12.1–37.2)	24.8 (23.1–26.5)	0.61
35–49	24.4 (13.8–39.6)	29.2 (27.5–30.9)	1.07
50–64	51.1 (36.3–65.8)	25.4 (24.0–26.9)	5.83
65–79	2.2 (0.3–15.1)	20.7 (19.4–22.0)	4.53
Marital status^3^, % (95% CI)			
Married and living with partner	11.1 (2.2–20.0)	60.8 (58.5–63.2)	26.87
Married and not living with partner/Single/Divorced/Widowed	88.9 (80.0–97.8)	39.2 (36.9–41.6)	10.18
Educational level^4^, % (95% CI)			
Low	2.3 (0.0–6.8)	35.0 (32.6–37.6)	6.90
Medium	36.4 (22.7–52.3)	50.8 (48.7–52.9)	2.89
High	61.4 (47.7–45.0)	14.2 (12.6–16.0)	13.51

*CI* confidence interval, *CIDI* Composite International Diagnostic Interview, *Cohen’s d* effect size computed for groups with different sample size, by adjusting the calculation of the pooled standard deviation with weights for the sample sizes

^1^Clinical Sample at Charité - Universitätsmedizin Berlin: 2018–2019; *n* = 45 re-participants based on *n* = 60 patients who participated in inpatient treatment with the Cognitive Behavioral Analysis System of Psychotherapy (CBASP [40]), age range: 24–66 years

^2^German Health Interview and Examination Survey for Adults, mental health module (DEGS1-MH): 2009–2012, weighted for population structure as of 31st December 2010; age range: 18–79 years; *n* = 4408 with full CIDI mood disorders section

^3^In DEGS1-MH, *n* = 32 subjects did not provide information on marital status

^4^Categorization according to the Comparative Analysis of Social Mobility in Industrial Nations (CASMIN) scale. There were missing values for *n* = 1 subject in the clinical sample and *n* = 17 participants in the DEGS1-MH sample

### Chronic depression at a population level

Among cases with lifetime MDD in DEGS1-MH (14.5%), 18.2% reported a maximum episode duration of at least 2 years, and 15.4% fulfilled the diagnostic criteria of concurrent DD (without considering criterion D). Overall, 36.5% of cases with a lifetime CIDI diagnosis of MDD were classified as chronic MDD cases; the remaining 63.5% were categorized as NCMDD cases. In addition to chronic MDD, PDD also comprised subjects with solely lifetime DD (1.3%, without considering criterion D).

### Characteristics of chronic depression in a clinical sample and at a population level

PDD cases from the clinical sample had a significantly earlier disease onset than cases with PDD and NCMDD in DEGS1-MH (age of disorder onset < 21 years: 73.3% vs. 24.7% vs. 32.2%, see Table *2*). Suicidality (thoughts of death/suicide, or having suicide plans/attempts) was reported more often by PDD cases in the clinical sample than by PDD or NCMDD cases in the DEGS1-MH sample (95.5% vs. 86.4% vs. 86.2%), as was attempted suicide (36.4% vs. 16.2% vs. 11.7%), but the significance of these differences remains unclear with one exception: the proportion of PDD patients in the clinical sample that attempted suicide was more than three times greater than the proportion of NCMDD cases in DEGS1-MH. Regarding self-reported depression course, PDD cases differed significantly from NCMDD cases in DEGS1-MH (*p* < 0.001). Both PDD groups showed significantly higher rates of a chronic course of a single episode compared with NCMDD cases (25.0 and 24.3% vs. 5.9%). Furthermore, a significantly higher proportion of clinical PDD patients showed a persistent depressive course compared with PDD and NCMDD DEGS1-MH cases (50.0% vs. 24.6% vs. 2.0%), and a smaller frequency of recurring episodes (2.3% vs. 20.9% vs. 55.1%; significant difference only for clinical PDD patients compared with NCMDD cases). Accordingly, cases with PDD in DEGS1-MH reported a significantly higher mean number of episodes in total (13.7) compared with both clinical PDD patients (2.8) and NCMDD cases in DEGS1-MH (7.4, *p* < 0.001). Comorbid mental disorders seemed to be more pronounced among cases with PDD and NCMDD in DEGS1-MH compared with the clinical sample, but the significance of these differences remains unclear. There was a trend for higher comorbidity among PDD cases than among NCMDD cases in DEGS1-MH (*p* = 0.071)*.* The prevalence of current depressive symptoms was highest among the clinical PDD patients (PHQ-9 ≥ 10: 66.7%), and significantly higher among PDD cases compared with NCMDD cases in DEGS1-MH (44.9% vs. 18.6%, *p* < 0.001). Furthermore, clinical PDD cases showed a significantly higher treatment resistance than PDD and NCMDD cases in DEGS1-MH, in terms of the proportion of cases reporting at least two psychotherapeutic treatments (90.9% vs. 2.7% vs. 0.9%) or antidepressant medications (81.0% vs. 9.1% vs. 12.2%) during the lifetime. Most PDD and NCMDD cases in DEGS1-MH reported no psychotherapeutic treatment (87.2 and 92.6%) or antidepressant medication (79.5 and 75.1%).

**Table 2:** Characteristics of cases with (non-)chronic depression during the lifetime in clinical and population-based samples

	Cases with PDD in clinical sample^1^ (*n* = 45)	Cases with PDD in DEGS1-MH^2^ (*n* = 179)	Cases with NCMDD in DEGS1-MH (*n* = 205)	*p*-value^5^
Age of disorder onset < 21 years^3^, % (95% CI)	73.3 (58.0–84.5)	24.7 (18.2–32.6)	32.2 (23.8–41.9)	0.192
Thoughts of death/suicide, or suicide plans/attempts^3^, % (95% CI)	95.5 (82.8–98.9)	86.4 (78.7–91.6)	86.2 (77.6–91.8)	0.960
Attempted suicide^3^, % (95% CI)	36.4 (23.2–52.0)	16.2 (9.2–26.8)	11.7 (6.9–19.3)	0.393
Self-reported depression course^3^, % (95% CI)				**< 0.001**
Single episode, remitted	–	**2.3 (0.9–5.4)**	**17.7 (10.4–28.3)**	
Single episode, acute	–	**5.7 (2.7–11.7)**	**4.0 (1.6–9.9)**	
Recurring episodes	2.3 (0.3–15.5)	**20.9 (14.4–29.3)**	**55.1 (45.0–64.8)**	
Single episode, chronic course	25.0 (14.1–40.4)	**24.3 (14.9–36.9)**	**5.9 (3.1–10.9)**	
Persistent depressive course	50.0 (35.1–64.9)	**24.6 (16.9–34.4)**	**2.0 (0.5–7.9)**	
Double depression	15.9 (7.5–30.5)	**19.3 (12.4–28.8)**	**11.9 (6.4–21.3)**	
Other	6.8 (2.1–19.9)	**2.9 (0.9–9.4)**	**3.5 (1.1–9.9)**	
No. of episodes^3^, mean (95% CI)	2.8 (1.9–3.6)	**13.7 (8.4–19.0)**	**7.4 (5.1–9.7)**	**< 0.001**
No. of comorbid mental disorders (lifetime)^4^, % (95% CI)				0.071
0	46.7 (32.2–61.7)	28.7 (20.8–38.2)	36.9 (28.0–46.7)	
1	33.3 (20.8–48.8)	28.9 (21.7–37.5)	37.4 (29.0–46.8)	
2	13.3 (5.9–27.3)	31.0 (21.8–42.0)	16.4 (10.5–24.8)	
≥ 3	6.7 (2.1–19.4)	11.4 (6.2–20.1)	9.3 (5.0–16.6)	
Current depressive symptoms (PHQ-9 ≥ 10), % (95% CI)	66.7 (51.2–79.2)	**44.9 (35.1–55.1)**	**18.6 (12.1–27.5)**	**< 0.001**
No. of psychotherapeutic treatments (lifetime)				0.271
0	–	87.2 (76.3–93.5)	92.6 (86.6–96.0)	
1	9.1 (2.8–2.6)	10.1 (4.7–20.3)	6.5 (3.3–12.6)	
≥ 2	90.9 (74.1–97.2)	2.7 (1.1–6.7)	0.9 (0.3–2.4)	
No. of antidepressant medications (lifetime)				0.674
0	2.4 (0.3–16.2)	79.5 (71.7–85.6)	75.1 (66.2–82.3)	
1	16.7 (7.9–31.8)	11.4 (7.4–17.1)	12.7 (7.8–20.1)	
≥ 2	81.0 (65.6–90.5)	9.1 (5.1–15.9)	12.2 (7.0–20.3)	

*CI* confidence interval, *PDD* persistent depressive disorder, *NCMDD* non-chronic major depressive disorder, *SCID I* Structured Clinical Interview for DSM-IV, *CIDI* Composite International Diagnostic Interview

^1^Clinical Sample at Charité - Universitätsmedizin Berlin: 2018–2019; *n* = 45 re-participants based on *n* = 60 patients who participated in inpatient treatment with the Cognitive Behavioral Analysis System of Psychotherapy (CBASP [40]), age range: 24–66 years

^2^German Health Interview and Examination Survey for Adults, mental health module (DEGS1-MH): 2009–2012, weighted for population structure as of 31st December 2010; age range: 18–79 years; *n* = 4408 with full CIDI mood disorders section

^3^Based on CIDI

^4^Based on SCID I or CIDI diagnoses of panic disorder, agoraphobia, generalized anxiety disorder, social phobia, specific phobias, obsessive–compulsive disorder, posttraumatic stress disorder, pain and somatoform disorders, substance abuse and dependence (excluding nicotine), anorexia nervosa, bulimia nervosa, and binge eating disorder

^5^*p*-value based on Rao–Scott chi-square test for categorical variables and based on Wilcoxon–Mann–Whitney test for continuous variables in DEGS1-MH. Bold type indicates significant differences between subjects with PDD and NCMDD in DEGS1-MH (local significance level α = 0.01)

### Health-related correlates of (non-)chronic depression at a population level

The associations of PDD vs. NCMDD with health-related correlates based on DEGS1-MH are shown in Tables 3 and 4. The risk of experiencing fair or poor self-rated health was significantly higher among PDD cases (36.8%) than among NCMDD cases (20.4%, odds ratio [OR] = 2.0, *p* = 0.041). Mean health-related quality of life (past 4 weeks) was lower among PDD cases for PCS (47.1 vs. 50.7, significant only for crude effect estimates, see Additional file 1) and MCS (34.5 vs. 43.8, β = − 8.2, *p* < 0.001). Accordingly, the mean number of days with activity limitations (past 4 weeks) owing to mental health problems was higher for PDD than for NCMDD (5.4 vs. 2.4, incidence rate ratio [IRR] = 2.6, *p* < 0.001). There was also a trend for more reported limitation days owing to physical health problems for PDD compared with NCMDD cases (5.3 vs. 3.1, IRR = 1.4, *p* = 0.091). There was also a higher risk of sick days during the past 12 months for PDD cases (34.2 vs. 14.8), but this was only significant in the unadjusted analysis (see Additional file 1). Indicators of health service use during the past 12 months showed higher utilization rates for PDD than for NCMDD cases for the mean number of outpatient psychiatric or psychotherapeutic contacts (5.7 vs. 1.7, IRR = 2.7, *p* = 0.006). There was also a trend for PDD cases to report a higher mean number of nights in hospital compared with NCMDD cases (3.9 vs. 0.9, IRR = 1.9, *p* = 0.065). The mean number of outpatient physician visits (4.3 vs. 3.6) was only significantly higher for PDD cases in the unadjusted analysis (see Additional file 1). Furthermore, somatic comorbidity was significantly higher for PDD vs. non-chronic cases. The risk of having one chronic condition (31.0% vs. 20.6%, relative risk ratio [RRR] = 2.8, *p* = 0.008) or at least two comorbid conditions (26.2% vs. 15.6%, RRR = 3.2, *p* = 0.004) was approximately 3-fold for PDD. In contrast, most NCMDD cases (63.9%) had no somatic comorbidity at all (vs. 42.8% of PDD cases).

**Table 3:** Health-related correlates in cases with PDD vs. NCMDD during the lifetime at a population level^1^

	PDD (*n* = 179)	NCMDD (*n* = 205)
Fair/poor self-rated health, % (95% CI)	**36.8 (28.8–45.6)**	**20.4 (13.8–29.0)**
Health-related quality of life (past 4 weeks), mean (95% CI)		
Physical component score	47.1 (44.9–49.4)	50.7 (48.8–52.7)
Mental component score	**34.5 (32.2–36.8)**	**43.8 (41.7–45.9)**
No. of days with activity limitations (past 4 weeks), mean (95% CI)		
Owing to mental health problems	**5.4 (4.1–6.7)**	**2.4 (1.2–3.6)**
Owing to physical health problems	5.3 (3.9–6.7)	3.1 (1.9–4.2)
No. of sick days (past 12 months), mean (95% CI)	34.2 (17.6–50.8)	14.8 (9.9–19.7)
No. of outpatient physician visits (past 12 months), mean (95% CI)	4.3 (3.4–5.2)	3.6 (2.9–4.2)
No. of outpatient psychiatric or psychotherapeutic contacts (past 12 months), mean (95% CI)	**5.7 (2.1–9.4)**	**1.7 (0.7–2.7)**
No. of hospital nights (past 12 months), mean (95% CI)	3.9 (1.2–6.6)	0.9 (0.5–1.3)
No. of chronic somatic conditions^2^, % (95% CI)		
0	42.8 (33.7–52.5)	63.9 (53.7–72.9)
1	**31.0 (22.9–40.4)**	**20.6 (13.6–30.0)**
2+	**26.2 (19.6–34.1)**	**15.6 (10.0–23.5)**

*CI* confidence interval, *PDD* persistent depressive disorder, *NCMDD* non-chronic major depressive disorder, *CIDI* Composite International Diagnostic Interview

^1^German Health Interview and Examination Survey for Adults, mental health module (DEGS1-MH): 2009–2012, weighted for population structure as of 31st December 2010; age range: 18–79 years; *n* = 4408 with full CIDI mood disorders section

^2^Myocardial infarction (lifetime), chronic heart failure (lifetime), stroke (lifetime), osteoarthritis (lifetime), rheumatoid arthritis (past 12 months), osteoporosis (lifetime), gout (past 12 months), bronchial asthma (past 12 months), cirrhosis of the liver (lifetime), hepatitis (past 12 months), gastric-duodenal ulcer (past 12 months), cancer (lifetime), Parkinson’s disease (lifetime), epilepsy (past 12 months), hypertension (past 12 months), dyslipidemia (past 12 months), renal failure (lifetime), and inflammatory bowel disease (12 months)

Bold type indicates significant associations between depression course (PDD vs. NCMDD) and health-related correlates (at local significance level α = 0.05, resulting from multiple (continuous outcome), multiple negative binomial (dichotomous outcome) or multinomial (multinomial outcome) regression analyses; see Table 4 for detailed statistical parameters)

**Table 4:** Effect estimates for health-related correlates in cases of PDD vs. NCMDD (ref.) during the lifetime^1^

		Effect estimate (95% CI)	*p*-value
Fair/poor self-rated health	OR	**2.0 (1.0–3.9)**	**0.041**
Health-related quality of life (past 4 weeks)			
Physical component score	β	−1.2 (-3.9 – -1.5)	0.374
Mental component score	β	**−8.2 (-11.5 – -4.9)**	**< 0.001**
No. of days with activity limitations (past 4 weeks)			
Owing to mental health problems	IRR	**2.6 (1.6–4.3)**	**< 0.001**
Owing to physical health problems	IRR	1.4 (0.9–2.1)	0.091
No. of sick days (past 12 months)	IRR	1.3 (0.9–1.8)	0.193
No. of outpatient physician contacts (past 12 months)	IRR	1.0 (0.8–1.3)	0.847
No. of outpatient psychiatric/psychotherapeutic contacts (past 12 months)	IRR	**2.7 (1.3–5.4)**	**0.006**
No. of hospital nights (past 12 months)	IRR	1.9 (1.0–3.8)	0.065
No. of chronic somatic conditions			
0		ref.	
1	RRR	**2.8 (1.3–5.8)**	**0.008**
2+	RRR	**3.2 (1.4–7.0)**	**0.004**

Regression models include health-related correlates as dependent variables and depression course (PDD vs. NCMDD) as the independent variable (reference: NCMDD). All analyses were adjusted for sex, age group, educational level, marital status, social support, chronic somatic conditions (except for analysis of the no. of chronic somatic conditions as an outcome variable), and PCS (except for analysis of PCS as an outcome variable). OR: Odds ratio from logistic regression; β: β coefficient from linear model; IRR: incidence rate ratio from negative binomial regression or zero-inflated negative binomial regression; RRR: relative risk ratio from multinomial logistic regression; p-value for testing the effect of depression course (test for OR/IRR/RRR = 1 or β = 0)

*CI* confidence interval, *PDD* persistent depressive disorder, *NCMDD* non-chronic major depressive disorder, *PCS* physical component score, *CIDI* Composite International Diagnostic Interview

^1^German Health Interview and Examination Survey for Adults, mental health module (DEGS1-MH): 2009–2012, weighted for population structure as of 12/31/2010; age range: 18–79; *n* = 4408 with full CIDI mood disorders section

Bold type indicates significant associations between depression course (PDD vs. NCMDD) and health-related correlates (at local significance level α = 0.05, resulting from multiple (continuous outcome), multiple negative binomial (dichotomous outcome) or multinomial (multinomial outcome) regression analyses)

### Post-hoc power analyses

Results from post-hoc power analyses with the help of G*Power 3 [51] suggest that that the present sample size of *n* = 429 individuals was sufficient for the detection of moderate effects (ω = 0.30) within a chi-square goodness-of-fit test comparing PDD vs. NCMDD in clinical and population-based samples for each health-related correlate and an error probability of α = 0.05, at the power level of 1.00 (see Table 2). Moreover, results from post-hoc power calculation suggest that the present sample size of *n* = 285 individuals was sufficient for the detection of moderate effects (*f*^2^ = 0.15) within a multiple regression design containing five predictors (PDD vs. NCMDD, age, sex, marital status, educational level) on each health-related correlate in a population-based sample, with an error probability of α = 0.05 and at the power level of 1.00 (see Table 4).

## Discussion

Based on a nationally representative sample of the general adult population in Germany, more than one-third (36.5%) of all subjects fulfilling MDD criteria showed a chronic depression course with maximum episode duration of at least 2 years and/or concurrent dysthymia at least once during the lifetime. This rate is slightly higher than previous international frequency estimates, which reported a chronic course for only 21 to 30% of depressed cases [12, 15–17]. This inconsistency can be explained by different definitions of chronic depression: previous prevalence based solely on episode duration, without considering MDD cases with double depression (i.e., MDD and DD).

### More severe PDD cases in the health care system

Overall, our estimated frequency for DEGS1-MH cases with MDD that had a chronic course during the lifetime (36.5%) was much lower than the proportion reported from national health insurance data (65%) [19]. However, previous findings show that among cases with CIDI-based MDD, 65.4% did not report any health service use for mental health problems [52]; and service use increased with depression severity [52]. Thus, particularly severe (and chronic) depression cases may eventually access the health care system, leading to higher proportions of chronic depression courses based on health insurance data [see 19] compared with frequency estimates for interview-based MDD cases at a population level.

Consequently, our comparisons of depression characteristics indicate that PDD cases in the health care system are more severely affected, since clinical sample PDD patients showed a pronounced long-term duration owing to earlier onset (73.3% vs. 24.7% with age onset ≤21 years) and significantly higher rates of treatment resistance (81.0% vs. 9.1% reported at least two antidepressant medication trials) compared with interview-defined PDD cases at a population level, as well as a higher proportion of self-classified persistence of depressive course. Furthermore, the prevalence of attempted suicide during lifetime was higher among clinical PDD patients as compared to the DEGS1-MH PDD cases (but nonsignificant) and more than three times higher than among NCMDD cases.

Considering the existing literature, our results are in line with clinical findings. For instance, lifetime prevalence of treatment resistance for depression was 81.8% in patients with long-term depression vs. 60.7% in patients with depression lasting less than 2 years [14]. In terms of inpatient treatment, a lifetime prevalence of 24.1% for hospitalization owing to mental health problems has been reported for PDD patients compared to 12.1% for non-PDD patients [17]. Furthermore, the average duration of past inpatient treatment is longer for PDD cases [53]. Patients with PDD also have higher rates of suicidal attempts and suicidal thoughts and are more likely to have a higher frequency of treatment approaches in general and a longer disorder duration [22].

Early depression onset seems a particular marker of a more severe PDD course: 73% of our clinical PDD patients showed an early onset, whereas the proportion was much lower among interview-defined PDD cases in DEGS1-MH (24.7%); and there was no significant difference between PDD and NCMDD cases at a population level. Similarly, international findings are heterogeneous: one meta-analysis found a significant relationship between early onset of depression and chronicity of the disorder [54]. However, in a recent review of 17 studies directly comparing age of onset in PDD vs. non-PDD cases, half the studies reported earlier onset for chronic vs. non-chronic depression whereas the other half reported no difference [22].

Recent reviews found that patients with PDD more often have psychiatric comorbidities than those with non-PDD, particularly personality disorders but also axis I and somatic comorbidities [22]. However, differences between PDD and NCMDD cases in DEGS1-MH have only been observed by trend, and our clinical sample of PDD patients demonstrated even less comorbidity than interview-defined cases. This may be related to differences in the diagnostic tools (SCID I vs. CIDI). Additionally, personality disorders, which account for a large proportion of comorbidities in the reviews, were not assessed in both samples. However, interpersonal problems as indicated by the social functioning subscale of the SF-36 were significantly reduced among PDD cases as compared to NCMDD cases in DEGS1-MH (post hoc sensitivity analysis; PDD: M = 61.47, 95%CI = 55.89–67.05; NCMDD: M = 76.77, 95%CI = 72.67–80.86). Consequently, only minor and non-significant differences in mental comorbidity have been observed between interview-defined PDD and NCMDD cases.

### Higher disease burden for chronic vs. non-chronic depression

The comparison of interview-defined cases of PDD vs. NCMDD at a population level highlighted that several health-related correlates indicate higher individual and economic disease burden for chronic depression courses.

On the individual level, there was a remarkably higher prevalence of current depressive symptoms (as assessed by PHQ-9) among PDD cases than among NCMDD cases, as well as a higher mean number of depressive episodes (irrespective of episode severity or duration). Furthermore, higher levels of psychological and somatic comorbidity are in line with international findings on higher comorbidity rates [12, 14, 15, 18, 20, 22] and somatic morbidity [14, 15] for chronic depression courses. The present outcomes of worse self-rated health and reduced health-related quality of life for the MCS correspond to previous findings of reduced psychological well-being and health-related quality of life for individuals with chronic depression [12–14, 23].

Accordingly, chronic depression is associated with higher indirect economic costs: PDD cases showed a higher risk of experiencing limitation days owing to mental health problems than non-chronic cases. Our findings of higher rates of outpatient mental health care utilization and the trend for a higher mean number of nights in hospital also indicate higher direct costs for the national economy and correspond to previous research findings [55].

### Public health implications and future perspectives

Considering the growing frequency of depression and health care costs in Germany owing to working days lost, early retirement, and health service provision [8, 56–58], our data strongly support the relevance of PDD as a specific course of depressive disorders. As long-term PDD is often associated with higher treatment resistance [59] there is a chance that if an early and tailored treatment of PDD and its specific psychopathological characteristics (e.g. CBASP) is carried out, a positive shift towards a more positive course of the disease can be achieved.

However, self-reported utilization rates [see 52] correspond with reported international treatment gaps for mental disorders in general [3, 4, 6, 7]: most Germans with acute depression do not access mental health care. In addition, previous results indicate more frequent help-seeking with higher education [60]. The characteristics of our clinical sample also suggest that in particular PDD cases with lower educational levels do not seek help or receive (specialized) treatment: While international findings show that PDD is associated with low socioeconomic status [61], PDD cases in our clinical sample had a significantly higher education as compared to the general population (DEGS1-MH participants). This is important, as it raises the question of whether more educated patients are more willing to participate in a depression intervention, or more likely to be informed about specific treatment programs for PDD. If so, then PDD patients with lower education may be disadvantaged in this regard.

Moreover, findings from national health care data suggest that the validity of medical depression diagnoses are questionable, particularly in primary care [62], and that improving treatment targeting [63, 64] and treatment quality [19, 65–68] are desirable. In conclusion, these findings highlight the need for national public health initiatives in Germany to reduce barriers to accessing mental health care services in general and in individuals with low education in particular, to strengthen awareness using targeted information campaigns, and to improve the quality of medical recognition and specialized treatment provision for depression and its different courses.

There is thus a need to identify patients with PDD correctly and to tailor specific treatment strategies. Therefore, a focus on psychological characteristics [69, 70] is warranted, as the DSM-5 diagnosis of PDD is very likely a heterogeneous umbrella diagnosis. For example, different studies could differentiate PDD and non-PDD in terms of psychopathological features and social functioning (e.g., cognitive and affective reactivity [69, 70] and interpersonal behavior [71]. This is important for the development of new treatment approaches as well as for the empirical corroboration and refinement of existing treatment attempts. For instance, CBASP was specifically developed for the treatment of PDD [40]. CBASP particularly considers psychopathological features of PDD such as an early onset due to childhood maltreatment and interpersonal withdrawal and avoidance. Evidence for the effectiveness of CBASP is encouraging (e.g. [72]), especially in patients with childhood maltreatment [73]. There is also evidence that the improvement of interpersonal behavior through CBASP is associated with symptom reduction, thus providing an important treatment target for PDD [74]. In this regard, CBASP proved to be more effective than less specific psychotherapeutic treatments [75, 76].

### Limitations

In interpreting the findings of this study some potential limitations should be considered, such as the study design, response and reporting bias, and construct overlap.

The small number of PDD cases in both the clinical sample and the DEGS1-MH sample may have reduced the accuracy of the frequency and mean estimates. Thus, significant differences between the samples may not have been detected using non-overlapping 95% CI.

Comparisons between PDD cases of DEGS1-MH and cases of the clinical sample are limited for several reasons. Particularly severe and chronic depression cases may be underrepresented in DEGS1-MH owing to the exclusion of institutionalized subjects, selective non-responses of less healthy individuals, and the inclusion of participants with private health insurance as well as some longitudinal participants (with a potentially greater probability of re-participation among healthier persons) [33–35]. Moreover, we found that clinical PDD patients had a higher educational level than DEGS1-MH participants. This also limits the group comparison. However, it could indicate that patients with PDD and a higher educational level have easier access to specified treatment programs. The comparison between the DEGS1-MH and clinical samples is further limited in terms of psychological comorbidity, owing to the use of different diagnostic tools (CIDI vs. SCID I). Furthermore, recall bias may have been more pronounced for DEGS1-MH cases, since PDD was defined on the basis of lifetime information, whereas the clinical sample only included patients diagnosed by PDD within the last 6 years. Thus, recall bias may have led to an underestimation of depression characteristics particularly among DEGS1-MH cases, e.g. with regard to treatment resistance and a history of suicidality. Furthermore, recall bias, varying diagnostic accuracy, and participants’ reporting bias may also have led to the underestimation of comorbidity and chronic depression course during the lifetime in both surveys, particularly among male and older participants [64, 77].

In DEGS1-MH, the small number of PDD and NCMDD cases may also have led to low statistical power for detecting the effects of depression course on health-related outcomes. Furthermore, time lags between the core DEGS1 survey and its mental health supplement may have led to an underestimation of associations between PDD/NCMDD and health-related correlates, as well as differing reference time frames for CIDI-based depression course during the lifetime and outcome variables (e.g., health service utilization during the past 12 months). However, construct overlap between depressive symptoms and the examined outcome measures (e.g., SF-36 and limitation days) may have led to the overestimation of associations.

## Conclusions

Finally, a chronic course of depression is challenging for both patients and practitioners. However, a knowledge gap remains regarding the lifetime characteristics and correlates of chronic depression and the reliability of the PDD concept itself.

By combining clinical and epidemiological perspectives, our study permitted a comparison of standardized characteristics of PDD among clinical vs. non-clinical cases and therefore extends existing knowledge about PDD. Our data suggest that the distinction between chronic and non-chronic depression proposed for DSM-5, in the form of PDD, is warranted. In particular, early onset depression, attempted suicide, self-classification as persistent depressive course, and treatment resistance are suggested as markers of more severe and chronic depression courses.

Furthermore, health-related correlates of PDD vs. non-chronic depression were compared at a population level. Thus, the associated individual and economic disease burden was evaluated for the general population in Germany for the first time. At a population level, chronic depression is associated with a remarkably higher disease burden than non-chronic courses, indicating enormous direct and indirect costs of chronic depression for the national economy and emphasizing its public health relevancy. In conclusion, these findings can inform the planning and targeting of prevention and health services. They highlight the need to further reduce barriers to accessing mental health care, improve awareness of different depression courses among health professionals, and implement specific treatment concepts for chronic depression.

## Supplementary information

**Additional file 1.** Unadjusted effect estimates for health-related correlates in cases of PDD vs. NCMDD (ref.) during the lifetime.

## Data Availability

Population-based data from the German health monitoring program that support the findings of this study are available from the Robert Koch Institute (RKI) but restrictions apply to the availability of these data, which were used under license for the current study and so are not publicly available. The data set cannot be made publicly available because informed consent from study participants did not cover public deposition of data. However, a minimal data set is archived in the Health Monitoring Research Data Centre at the RKI and can be accessed by all interested researchers. On-site access to the data set is possible at the Secure Data Centre of the RKI’s Health Monitoring Research Data Centre. Requests should be submitted to the Health Monitoring Research Data Centre, Robert Koch Institute, Berlin, Germany (Email: fdz@rki.de). The patient data from the clinical population are available from the Charité – Universitätsmedizin Berlin. The data set cannot be made publicly available because informed consent from study participants did not cover public deposition of data.

## Abbreviations

95% CI: 95% confidence interval
CASMIN: Comparative Analysis of Social Mobility in Industrial Nations
CBASP: Cognitive Behavioral Analysis System of Psychotherapy
CIDI: Composite International Diagnostic Interview
DD: dysthymic disorder
DEGS1: German Health Interview and Examination Survey for Adults
DEGS1-MH: Mental health module of DEGS1
DSM-IV-TR: Diagnostic and Statistical Manual of Mental Disorders, 4th Edition, Text Revision
IRR: incidence rate ratio
MCS: mental component score
MDD: major depressive disorder
NCMDD: non-chronic major depressive disorder
OR: odds ratio
PCS: physical component score
PDD: persistent depressive disorder
PHQ-9: Patient Health Questionnaire-9
RRR: relative risk ratio
SCID I: Structured Clinical Interview for DSM-IV
SF-36: Short Form Health Survey-36
