# Supplementary material for: Persistent depressive disorder across the adult lifespan: results from clinical and population-based surveys in Germany
Source: BMC Psychiatry. 2020 Feb 10;20:58. doi: 10.1186/s12888-020-2460-5 (PMC7011512; doi:10.1186/s12888-020-2460-5)
Supplement: Supplementary file 1 — Additional file 1. Unadjusted effect estimates for health-related correlates in cases of PDD vs. NCMDD (ref.) during the lifetime. [file 12888_2020_2460_MOESM1_ESM.docx]

**Persistent depressive disorder across the adult lifespan: results from clinical and population-based surveys in Germany**

Julia Nübel^1^, Anne Guhn^2^, Susanne Müllender^1^, Hong Duyen Le^1^, Caroline Cohrdes^1^* and Stephan Köhler^2^

^1^ Department of Epidemiology and Health Monitoring, Unit 26 Mental Health, Robert Koch Institute, PO Box 650261, D13302 Berlin, Germany

^2^ Department of Psychiatry and Psychotherapy, Charité – Universitätsmedizin Berlin, Campus Mitte, Charitéplatz 1, 10117 Berlin, Germany

* Correspondence: CohrdesC@rki.de

**Additional file 1**

Unadjusted effect estimates for health-related correlates in cases of PDD vs. NCMDD (ref.) during the lifetime^1^

|  |  | **Effect estimate**  **(95% CI)** | **p-value** |
| --- | --- | --- | --- |
| **Fair/poor self-rated health** | OR | **2.3 (1.3**–**4.1)** | **0.006** |
| **Health-related quality of life (past 4 weeks)** |  |  |  |
| Physical component score | β | **-3.6 (-6.7**–**-0.5)** | **0.023** |
| Mental component score | β | **-9.3 (-12.6**–**-6.0)** | **<0.001** |
| **No. of days with activity limitations (past 4 weeks)** |  |  |  |
| Owing to mental health problems | IRR | **2.8 (1.8**–**4.3)** | **<0.001** |
| Owing to physical health problems | IRR | **1.9 (1.3**–**2.9)** | **0.002** |
| **No. of sick days (past 12 months)** | IRR | **2.3 (1.6**–**3.3)** | **<0.001** |
| **No. of outpatient physician contacts (past 12 months)** | IRR | **1.3 (1.0**–**1.6)** | **0.016** |
| **No. of outpatient psychiatric/psychotherapeutic contacts (past 12 months)** | IRR | **2.8 (1.5**–**5.2)** | **0.001** |
| **No. of hospital nights (past 12 months)** | IRR | **2.5 (1.4**–**4.7)** | **0.003** |
| **No. of chronic somatic conditions** |  |  |  |
| 0 |  | ref. |  |
| 1 | RRR | **2.2 (1.1**–**4.4)** | **0.019** |
| 2+ | RRR | **2.5 (1.3**–**4.9)** | **0.007** |

^1^ German Health Interview and Examination Survey for Adults, mental health module (DEGS1-MH): 2009-2012, weighted for population structure as of 12/31/2010; age range: 18-79; n = 4408 with full CIDI mood disorders section.

Models include health related correlate as dependent and depression course (PDD vs. NCMDD) as independent variable (reference: NCMDD). OR: Odds ratio from logistic regression; β: β coefficient from linear model; IRR: incidence rate ratio from negative binomial regression or zero-inflated negative binomial regression; RRR: relative risk ratio from multinomial logistic regression; p-value for testing an effect of depression course (test for OR/IRR/RRR=1 or β=0).

Bold type significant associations between the depression course (PDD vs. NCMDD) and health-related correlates (at local significance level α = 0.05, resulting from multiple (continuous outcome), multiple negative binomial (dichotomous outcome) or multinomial (multinomial outcome) regression analyses.

CI: confidence interval; PDD: persistent depressive disorder; NCMDD: non-chronic major depressive disorder; PCS: physical component score; CIDI: Composite International Diagnostic Interview.
